# RELIABILITY AND VALIDITY OF THE KNOWLEDGE OF PERSON-CENTERED BEHAVIORAL APPROACHES FOR BPSD

**DOI:** 10.1093/geroni/igz038.2064

**Published:** 2019-11-08

**Authors:** Kimberly Van Haitsma, Caroline Madrigal, Ann M Kolanowski, Barb Resnick, Beth Galik, Jeanette Ellis, Karen Eshraghi

**Affiliations:** 1 penn State, University Park, Pennsylvania, United States; 2 University of Maryland, Baltimore, Maryland, United States; 3 Penn State, Baltimore, Maryland, United States

## Abstract

How behavioral and psychological symptoms of dementia (BPSD) are understood and managed is important to person-centered care. No knowledge tests associated with dementia specifically address staff knowledge of person-centered behavioral approaches to BPSD. The Knowledge of Person-Centered Behavioral Approaches for BPSD Test was developed by our team to fill that gap. We tested the reliability and validity of this measure using a Rasch analysis and additional psychometric testing. 1,071 nurses from 35 nursing homes participated in the study. Reliability was evident based on an item separation of 11.00 and item reliability of 0.99. Construct validity was evident in that all of the items fit the model with INFIT and OUTFIT statistics (0.6-1.4). Associations between test scores and observed positive and negative care interactions (r=.38, p=.03; r=-.26, p=.12), person-centered care approaches (r=-.25, p=.15), and resistance to care (r=-.31, p=.07) will be examined and implications for person-centered care discussed.

